# Minimal Clinically Important Differences in EQ-5D-5L Index and VAS after a Respiratory Muscle Training Program in Individuals Experiencing Long-Term Post-COVID-19 Symptoms

**DOI:** 10.3390/biomedicines11092522

**Published:** 2023-09-13

**Authors:** Tamara del Corral, Raúl Fabero-Garrido, Gustavo Plaza-Manzano, Marcos José Navarro-Santana, César Fernández-de-las-Peñas, Ibai López-de-Uralde-Villanueva

**Affiliations:** 1 Department of Radiology, Rehabilitation and Physiotherapy, Faculty of Nursing, Physiotherapy and Podiatry, Universidad Complutense de Madrid (UCM), 28040 Madrid, Spain; tamaradelcorral@gmail.com (T.d.C.); rfabero@ucm.es (R.F.-G.); marconav@ucm.es (M.J.N.-S.); ibai.uralde@gmail.com (I.L.-d.-U.-V.); 2 Instituto de Investigación Sanitaria del Hospital Clínico San Carlos (IdISSC), 28040 Madrid, Spain; 3 Department of Physical Therapy, Occupational Therapy, Rehabilitation and Physical Medicine, Universidad Rey Juan Carlos, 28922 Alcorcón, Spain; cesar.fernandez@urjc.es

**Keywords:** SARS-CoV-2, minimal clinically important difference, health-related quality of life, euroqol-5D-5L, responsiveness

## Abstract

The primary aim of this study was to determine the minimal clinically important difference (MCID) for the EuroQol-5D questionnaire (EQ-5D-5L) index and visual analogic scale (VAS) in individuals experiencing long-term post-COVID-19 symptoms. In addition, it was pretended to determine which variable discriminates better and to compare changes between individuals classified by the MCID. Design: Secondary analysis of a randomized controlled trial involving 42 individuals who underwent an 8-week intervention in a respiratory muscle training program. Results: A change of at least 0.262 and 7.5 for the EQ-5D-5L index and VAS represented the MCID, respectively. Only the EQ-5D-5L VAS showed acceptable discrimination between individuals who were classified as “improved” and those classified as “stable/not improved” (area under the curve = 0.78), although with a low Youden index (Youden index, 0.39; sensitivity, 46.2%; specificity, 93.1%). Those individuals who exceeded the established MCID for EQ-5D-5L VAS had significantly greater improvements in inspiratory muscle function, exercise tolerance, and peripheral muscle strength compared to participants classified as “stable/not improved”. Conclusions: Only the EQ-5D-5L VAS, especially when MCID was exceeded, showed an acceptable discriminative ability to evaluate the efficacy of an intervention in individuals with long-term post-COVID-19 symptoms.

## 1. Introduction

The coronavirus disease 2019 (COVID-19) is an illness caused by SARS-COV-2 and responsible for a global pandemic. It has become one of the most pressing global health and socio-economic issues of our time. Since its emergence in late 2019, the pandemic has affected millions of people across the globe, causing widespread illness, mortality, and economic disruption [1]. While many individuals recover fully within a few weeks, there is increasing evidence that some patients experience persistent symptoms for months after the initial infection, including fatigue, dyspnea, sleep disorders, and myalgia [2]. Patients with post-COVID-19 symptoms exhibit alterations in respiratory muscle function [3], a hyperventilation response, and decreased exercise tolerance [4]. These long-term symptoms caused by COVID-19 can lead to sedentary behavior and a lower health-related quality of life.

Clinical studies frequently report treatment effects as a statistically significant difference. However, a statistically significant change may not necessarily reflect a meaningful clinical change. It is becoming increasingly recognized that evaluating the clinical benefit of treatment from the patient’s standpoint is crucial. Furthermore, it is necessary to evaluate the ability of the outcome measure to identify clinical changes and to develop methods to interpret the degree of the observed change [5]. The minimal clinically important difference (MCID) is defined as “the smallest difference in score that patients perceive as beneficial and which would mandate a change in the patient’s management” and adds clinical relevance and the patient’s perspective to the observed change. MCID of health-related quality of life has been previously reported from 0.03 to 0.52 using the EuroQol-5D-5L (EQ-5D-5L) index [6,7,8,9,10] and from 6.5 to 8.2 using the EQ-5D-5L visual analogue scale (VAS) [7,9,11]. The variation in the MCID is due to heterogeneity between studies, which means that extrapolating the results to populations with long-term post-COVID-19 symptoms could not be reliable. The MCID has not been determined for health-related quality of life in individuals with long-term post-COVID-19 symptoms. Therefore, the changes over time experienced by patients with long-term post-COVID-19 symptoms are difficult to interpret. The MCID can help researchers and clinicians interpret whether the magnitude of the observed change represents a relevant improvement after a respiratory muscle training (RMT) program in individuals with long-term post-COVID-19 symptoms.

Thus, the present study aimed to determine MCIDs for health-related quality of life (EQ-5D-5L index and VAS) in individuals with long-term post-COVID-19 symptoms. Secondarily, the study aimed to determine which of the EQ-5D-5L variables has a higher discriminatory capacity and to compare the changes with those found between individuals who exceed the MCID and those who do not in inspiratory muscle function, physical function (exercise tolerance and peripheral muscle strength), and pulmonary function.

## 2. Materials and Methods

### 2.1. Study Design

This study was a secondary analysis of data from a previously conducted randomized controlled trial (RCT; trial registration number: NCT04734561) with the primary aim of evaluating the effects of a home-based respiratory muscle training program on quality of life and exercise tolerance in individuals with long-term post-COVID-19 symptoms [12]. This RCT was a parallel, double-blind study in which participants were randomly assigned to one of the following groups: (1) inspiratory muscle training; (2) inspiratory and expiratory muscle training; (3) sham inspiratory muscle training; and (4) sham inspiratory and expiratory muscle training. All groups performed a training program consisting of 40 min/day (20 min sessions twice a day), 6 days per week, for 8 weeks. Clinical assessments were performed at baseline, 4 weeks and 8 weeks.

For this secondary analysis, data from the two real training groups was reanalyzed. To be included, participants had to complete their baseline and 8 weeks assessment. Thus, a total of 42 individuals with long-term post-COVID-19 symptoms were analyzed. According to the criterion considered by Cohen, an effect size of at least 0.5 is the minimum effect size to detect clinically relevant differences [13]. Thus, the estimated sample size calculation for a paired *t*-test with an α of 5%, a power of 80%, two-tailed, and an expected effect size of at least 0.5 is 34 individuals. Consequently, the analysis of 42 individuals could be considered acceptable for determining the MCID for health-related quality of life outcomes.

### 2.2. Participants

The trial enrolled people aged 18 years or older with post-COVID-19 symptoms (including fatigue and dyspnea) for at least 3 months after COVID-19 diagnosis. In addition, only individuals who had performed the real respiratory muscle training program were included in the current analysis to avoid a possible bias in the estimation of MCID due to the inclusion of individuals who performed sham training. Individuals with any of the following criteria were excluded from participation in the study: (1) a diagnosis of progressive respiratory, neuromuscular, or neurological disorders; (2) psychiatric/cognitive disorder or pathology affecting their ability to cooperate; (3) presence of any contraindication to undergoing respiratory muscle training, including no internet access; and (4) previous participation in a rehabilitation program to improve their long-term post-COVID-19 symptoms.

### 2.3. Outcome Measures

- Health-related quality of life: The EQ-5D-5L questionnaire was employed [14]. An index score is provided, ranging from 0 (death) to 1 (full health). Participants rated their current overall health on a VAS ranging from 0 (worst imaginable health) to 100 (best imaginable health).
- Inspiratory muscle function: Inspiratory muscle strength was assessed by the maximum static inspiratory pressure (MIP). Specifically, MIPs were recorded using a digital mouth pressure meter (MicroRPM^TM^; Carefusion, San Diego, CA, USA), according to the American Thoracic Society/European Respiratory Society (ATS/ERS) guidelines [15].
- Inspiratory muscle endurance (IME) was measured during a constant load breathing test using the POWERbreathe KH1^©^ device (POWERbreathe International Ltd., Southam, UK), following the instructions established in a previously published protocol [16].
- Exercise tolerance: The Ruffier test was employed [17]. Heart rate (HR) was recorded at 3 different times: (1) after 1 min of rest (HR_0_); (2) immediately after completing the 30 squats (HR_1_); and (3) after 1 min of recovery (HR_2_). Exercise tolerance was determined as follows: ((HR_0_ + HR_1_ + HR_2_) − 200)/10.
- Peripheral muscle strength: Lower and upper extremity muscle strength was assessed using the 1 min sit-to-stand test (1 min STS) [18] and the hand grip strength (Jamar^®^ hand-held dynamometer, Patterson Medical, IL, USA) [19], respectively.
- Lung function: Forced spirometry was assessed using a portable spirometer (Spirobank II USB^®^, MIR, Rome, Italy) [20].

### 2.4. Anchor Outcome

The MCID was determined using the 15-point global rating of change (GROC) ordinal scale [21], ranging from −7 (“much worse”) to 7 (“much better”). Participants answered the following question according to the GROC scale: “Compared to the first assessment/visit, how much change do you perceive after respiratory muscle training in your overall health status (including performance of activities of daily living, efforts/fatigue and/or dyspnea)?”.

### 2.5. Data Analysis

Analyses were performed with SPSS version 27.0 (SPSS Inc., Chicago, IL, USA), and statistical significance was set at 5% (*p* < 0.05). According to hypothesis tests and the central limit theorem [22], a normal distribution of the outcomes was assumed.

Differences in health-related quality of life between pre- and post-training outcomes were assessed using a paired samples *t*-test. In addition, effect sizes were calculated (small, *d* < 0.5; medium, *d* = 0.50–0.79; or large, *d* ≥ 0.8) [13].

The anchor-based method was used to determine the MCID. Concretely, a ROC curve analysis was performed using the GROC scale as an external criterion to determine the optimal cut-off value for the health-related quality of life variables (continuous variables) that corresponded with the least misclassification for discriminating between individuals who had improved and those who were unchanged or deteriorated. According to GROC scores, participants were dichotomized into 2 groups to calculate the MCID: (1) Stable/not improved (GROC = +3 or less; transformed for the criterion variable to 0); and (2) Improved (GROC = +4 or more; transformed for the criterion variable to 1). Traditionally, a cut-off point of +4 has been considered to determine the MCID [21,23]. Thus, the ROC curve analysis allows us to determine the minimum quantitative improvement in the health-related quality of life variables necessary to identify with greater sensitivity and specificity the individuals who improved.

Group comparisons between individuals with and without a change greater than the MCID in health-related quality of life outcomes were performed using non-parametric tests due to the sample size (sample size ≤ 14 individuals in one of the groups established according to the MCID for each variable). Mann–Whitney U test was used to detect between-group differences at baseline, post-training, and Δpre-post. The Wilcoxon test was used to assess within-group differences. The magnitude of the differences was calculated using *r* effect size: small (<0.3), medium (0.30–0.5), or large (>0.5) [24].

## 3. Results

Forty-two (n = 42) individuals with long-term post-COVID-19 symptoms constituted the study sample (71% female, age: 48 ± 9 years), whose mean duration of symptoms since COVID-19 diagnosis was 354.2 ± 77.5 days. All clinical characteristics of the sample can be observed in the original RCT study [12]. In addition, no adverse effects were reported, and adherence to the training program was optimal (>95% of scheduled sessions).

### 3.1. Minimal Clinically Important Difference

According to the GROC scores, 69% of participants were classified as “improved” and 31% as “stable/not improved”. After 8 weeks of respiratory muscle training, a large and statistically significant increase in health-related quality of life outcomes was observed in the whole sample (ΔEQ-5D-5L, index = 0.192 ± 0.165 (*p* < 0.001; *d* = 0.88); ΔEQ-5D-5L, VAS = 17.7 ± 12.71 (*p* < 0.001; *d* = 1.11)). Table 1 shows the descriptive statistics for change in health-related quality of life as well as the multiple comparisons between the group categorized as “improved” and the group categorized as “stable/not improved”.

**Figure 1:**
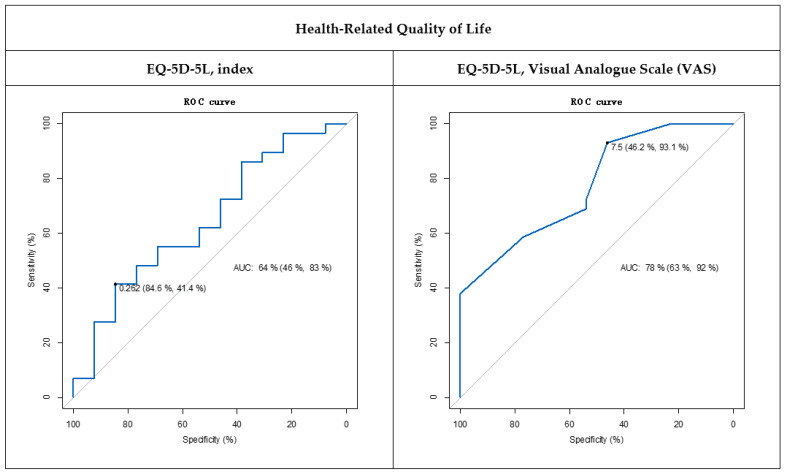
Receiver operating characteristic (ROC) curves (blue line) for health-related quality of life. Values expressed as: AUC (95% CI), Area Under the Curve (95% Confidence Interval); MCID (Sen, Spe), Minimal Clinically Important Difference (Sensitivity, Specificity). Gray line: a diagonal line corresponding to random chance.

According to the ROC analysis (see Table 2), only EQ-5D-5L VAS obtained acceptable discrimination between individuals who self-classified as “improved” and “stable/not improved” (AUC = 0.78; Figure 1). However, it is important to note that the Youden index for the EQ-5D-5L VAS was lower than 0.5 (Youden index = 0.39), because the proposed MCID was very specific but not very sensitive. ROC curve analysis established that a change of 0.262 (sensitivity = 84.6%; specificity = 41.4%) and 7.5 (sensitivity = 46.2%; specificity = 93.1%) represents a clinically significant improvement in the EQ-5D-5L index and EQ-5D-5L VAS, respectively. Thus, assuming 0.262 or 7.5 predicted as MCID for the EQ-5D-5L index and EQ-5D-5L VAS, 53.8% or 6.9% of participants who classified themselves as “stable/not improved” were misclassified as “improved,” respectively.

### 3.2. Individuals with a Change above MCID vs. Those with a Change below MCID

Multiple comparisons between individuals with and without a change greater than MCID in the outcomes examined can be seen in Table 3. Individuals with a change above the MCID set for the EQ-5D-5L index and the EQ-5D-5L VAS showed a moderate and statistically significant increase in inspiratory muscle function (MIP and IME) compared to those with a change below the MCID (*p* < 0.01; *r* ≥ 0.44), except for inspiratory muscle endurance, where there was no difference between individuals who exceeded the MCID of the EQ-5D-5L index and those who did not (*p* > 0.05; *r* = 0.27).

Individuals with a change greater than the MCID set for the EQ-5D-5L index showed a medium and statistically significant increase in handgrip (*r* = 0.44) but not for exercise tolerance (*p* = 0.759; *r* = 0.05) and 1 min STS (n of squats) (*p* = 0.092, *r* = 0.26) compared to those who did not exceed the MCID. Patients that exceeded the MCID for EQ-5D-5L, VAS showed a significant medium effect for exercise tolerance (*r* = 0.33) and 1 min STS (*r* = 0.35) but not for handgrip (*p* = 0.898; *r* = 0.02) compared to those who did not exceed the MCID. Individuals with a change greater than the MCID set for the EQ-5D-5L index and VAS did not show any significant improvement in lung function compared to those who did not exceed the MCID.

## 4. Discussion

The study of the MCID for the EQ-5D-5L has increased in recent years as it favors patient-based clinical decision making [10]. However, this is the first study to present the MCIDs for the health-related quality of life of individuals experiencing long-term post-COVID-19 symptoms after completing a respiratory muscle training program. Employing an anchor-based approach, an increase in the EQ-5D-5L index and EQ-5D-5L VAS of 0.262 and 7.5, respectively, could be deemed clinically significant for individuals experiencing long-term post-COVID-19 symptoms. Moreover, the EQ-5D-5L VAS reached the highest discriminative ability due to its superior metric properties in this population. The calculation of these MCIDs provides clinicians and researchers with a guide both for interpreting the significance of clinical changes in patients or intervention groups and for the calculation of sample sizes [10].

Similar to previous studies investigating the impact of individually tailored exercise on individuals experiencing long-term post-COVID-19 symptoms [25,26], both real respiratory muscle training groups showed a significant improvement in their health-related quality of life after an 8-week intervention, as measured by the EQ-5D-5L index and VAS. Respiratory muscle dysfunction has been linked to physical and functional decline in individuals with long-term post-COVID-19 symptoms [3]. Thus, respiratory muscle training could justify the observed quality of life improvements. The obtained effect sizes were large (*d* ≥ 0.8) for the health-related quality of life outcomes, indicating that the improvements observed could be considered clinically relevant [13]. Significant differences were detected for the EQ-5D-5L VAS but not for the EQ-5D-5L index between the “improved” group when compared to the “stable/not improved” group, which reinforces the higher discriminative ability detected for the EQ-5D-5L VAS. This is consistent with previous literature, which reports measurement disparities between the two components of the generic EQ-5D-5L instrument [27].

Although previous research demonstrated that the EQ-5D-5L questionnaire exhibits good psychometric properties in individuals experiencing long-term post-COVID-19 symptoms [28], only the EQ-5D-5L VAS showed an acceptable discrimination ability (AUC ≥ 0.7) between individuals who classified themselves as “improved” and “stable/not improved”. This finding is consistent with those found by Sakthong et al. [29] and Whynes et al. [27], demonstrating superior responsiveness of the EQ-5D-5L VAS over the EQ-5D-5L index in chronic conditions. However, other studies have concluded opposite results [30,31], arguing that the EQ-5D-5L VAS is more generic and responses are influenced not only by the patient’s state of health but also by personal characteristics such as psychological disposition, socioeconomic status, or disease condition [29,32]. Focusing on individuals with long-term post-COVID-19 symptoms, the EQ-5D VAS is known to detect more people with low quality of life compared to the EQ-5D-5L index [33]. This would confer on the EQ-5D-5L VAS a greater capacity to identify changes in the health status of individuals after an intervention, which would justify the results found in individuals with long-term post-COVID-19 symptoms.

To our knowledge, no previous study has identified the MCID for the EQ-5D-5L index or VAS in individuals with long-term post-COVID-19 symptoms. This limits our ability to discuss MCIDs with another researcher since they are disease-specific [10]. However, below are detailed MCIDs found in populations with similar characteristics using anchor-based methods from other investigations. The MCID of the EQ-5D-5L index in this study was 0.262, a far higher score compared to the MCID calculated for patients with diseases that share a major chronic fatigue component, such as chronic obstructive pulmonary disease [7], fibrotic interstitial lung disease [6], coronary heart disease [8], or lung cancer [9] (0.046; 0.05; 0.052; and 0.08, respectively). It is possible that the clinical instability reflected by individuals with long-term post-COVID-19 symptoms [1] could have an impact in terms of mobility, pain, anxiety, and activity performance, justifying the use of higher thresholds for MCID to detect those individuals who clinically improve their quality of life. However, the low metric properties detected for the EQ-5D-5L index (AUC < 0.7) preclude confidence in the selected cut-off point. The MCID for the EQ-5D-5L VAS was 7.5 in individuals with long-term post-COVID-19 symptoms, which is in agreement with previous research in other respiratory diseases, e.g., chronic obstructive pulmonary disease [7] or lung cancer [9] (6.5 and 7, respectively). The adequate discriminative ability detected for the EQ-5D-5L VAS further supports our results.

The MCID reflects the smallest difference in score, which patients perceive as beneficial, and should not be confused with the minimal detectable change (MDC) [10,34], which corresponds to the smallest change in score that can be detected beyond the random measurement error [34]. For a measurement in a given population, the MCID must be higher than the MDC to ensure that significant clinical changes in patients are detected and that these are not due to chance arising from measurement error. In patients with coronary heart disease, the MDC_95_ of the EQ-5D-5L index has been recently found to be 0.216 for individual levels and 0.025 for group levels [8], values that are below the MCID calculated in this investigation. The same applies to MDC_95_ for the EQ-5D-5L VAS, which has been found to be 2.32 and 4.5 in individuals with cervical intraepithelial neoplasia [35] and with axial spondylarthritis [36], respectively. This indicates that MCIDs could be discriminated from measurement error to reflect a real and significant change in individuals with long-term post-COVID-19 symptoms, even at the individual level, although calculation of the MDC in this specific population would be required.

Improvements in health-related quality of life seem to be consistent following an RMT program, not only in individuals with long-term post-COVID-19 symptoms [12,26], but also in other clinical conditions presenting respiratory muscle weakness [37,38]. In this line, in the study of Dall’Ago et al. [37] respiratory muscle strength and health-related quality of life improved up to one year after the intervention, reinforcing the plausibility that quality of life outcomes could be used as an indicator of success following RMT interventions. EQ-5D-5L VAS showed the highest LR+ (6.7) when a change ≥7.5 is produced, indicating a high probability of determining with high certainty that the individual would feel clinically better if he/she exceeded that value. All the other likelihood ratios calculated for the EQ-5D-5L index or VAS are too poor to ensure that patients will or will not obtain significant clinical improvements, so the most clinically useful scores should be those that exceed 7.5 points on the EQ-5D-5L VAS. These results support the use of VAS as a valid and reliable solution in the general assessment of health-related parameters [39], including health-related quality of life.

Group comparisons were successfully conducted between participants who exceeded the MCID and those who did not. This particular aspect stands out as one of the most noteworthy and robust aspects of this study. Thus, clinically meaningful improvements in health-related quality of life outcomes were correlated with improvements in inspiratory muscle function, exercise tolerance, and peripheral muscle strength in individuals with long-term post-COVID-19 symptoms. Increased exercise tolerance mediated by RMT is associated with a delay in the onset of respiratory metaboloreflex, as described by Fernandez-Rubio et al. [40]. Increased exercise tolerance could increase the individual’s functionality, activity levels, and probably peripheral muscle strength. Overall, RMT may have a positive biopsychosocial impact on the individual, which would justify the established associations with the EQ-5D-5L index and VAS. No associations have been found between the EQ-5D-5L and pulmonary function parameters since the effects of RMT at this level seem contradictory [12,38].

Certain limitations should be acknowledged in this study. First, this is a secondary analysis of a RCT, so it was not specifically designed to estimate the MCID of the EQ-5D-5L (index and/or VAS) nor to assess differences between individuals with and without a change above the MCID in these variables. According to the sample size calculation performed, the sample size is adequate to assess the objectives set out in this study. However, future studies with objectives focused on the calculation of the MCID are desirable. The relatively small number of participants without a change in GROC (31%) may have lowered the precision of calculating the specificity of the cut-points established. Considering that the MCID is specific for each population and even for each intervention modality [10], the generalization of these results should be performed with caution beyond the scope of this research.

Some clinical implications emerge from the present investigation. If an individual experiencing long-term post-COVID-19 symptoms undergoes RMT program and improves 7.5 points on the EQ-5D-5L VAS, it is highly likely that clinically relevant changes have occurred, signaling the success of the intervention. The establishment of the MCID allows the identification of clinically relevant improvements in health-related quality of life, which is useful for monitoring patients’ progress and checking whether they achieve the proposed objectives during rehabilitation. It also helps to evaluate the effectiveness of the interventions and the interpretation and understanding of the results of the investigations, relating the observed changes to what is relevant for the individual. Finally, the guidelines recommend relying on the MCID to establish a correct sample size within the research, acquiring a more patient-centered approach [10].

## 5. Conclusions

The results of this study showed that only the EQ-5D-5L VAS, but not the EQ-5D-5L index, acceptably discriminated against the efficacy of an intervention in individuals with long-term post-COVID-19 symptoms. The MCID for judging clinical change for the EQ-5D-5L index and VAS was set for values above 0.262 and 7.5, respectively. EQ-5D-5L VAS showed high accuracy when individuals exceeded the MCID. Individuals with long-term post-COVID-19 symptoms with a change greater than the MCID established for the EQ-5D-5L are expected to improve inspiratory muscle function, exercise tolerance, and peripheral muscle strength. More studies focused on the calculation of the MCID on health-related quality of life outcomes are needed in this population.

## Figures and Tables

**Table 1:** Descriptive statistics and between-group comparisons for change in health-related quality of life.

Outcome	Group	Mean ± SD; Median (IQR)			Within-Group Differences *p*-Value; *r* Effect-Size
		Baseline	Post-Training	ΔPre-Post	
EQ-5D-5L, index	Improved	0.608 ± 0.208 0.631 (0.470–0.782)	0.826 ± 0.138 0.830 (0.745–0.966)	0.219 ± 0.160 0.220 (0.090–0.322)	*p* < 0.001; *r* = 0.83
	Stable/not improved	0.554 ± 0.278 0.679 (0.365–0.792)	0.687 ± 0.297 0.786 (0.558–0.899)	0.133 ± 0.167 0.170 (0.019–0.225)	*p* = 0.017; *r* = 0.65
**Between-group differences for ΔPre-Post training** ***p*-value; *r* effect-size**				*p* = 0.138; *r* = 0.23	
EQ-5D-5L, VAS	Improved	57.6 ± 14.5 60 (50–65)	79.21 ± 13.16 75 (70–92.5)	21.6 ± 11.9 20 (10–30)	*p* < 0.001; *r* = 0.88
	Stable/not improved	53.8 ± 19.06 55 (35–67.5)	62.69 ± 15.63 60 (50–70)	8.8 ± 9.8 10 (5–15)	*p* = 0.014; *r* = 0.68
**Between-group differences for ΔPre-Post training** ***p*-value; *r* effect-size**				*p* = 0.004; *r* = 0.44	

Abbreviations: HRQoL, Health-Related Quality of Life; IQR, Inter Quartile Range; VAS, Visual Analogue Scale.

**Table 2:** Receiver operating characteristic (ROC) analysis results for health-related quality of life.

Outcome	MCID	AUC (95% CI)	Sensitivity	Specificity	Youden Index	LR+	LR−
HRQoL							
EQ-5D-5L, index	0.262	0.64 (0.46 to 0.83)	84.6	41.4	0.26	1.44	0.37
EQ-5D-5L, VAS	7.5	0.78 (0.63 to 0.92)	46.2	93.1	0.39	6.70	0.58

Abbreviations: AUC (95% CI), Area Under the Curve; CI, Confidence Interval; HRQoL, Health-Related Quality of Life; LR, Likelihood Ratio; MCID, Minimal Clinically Important Difference; VAS, Visual Analogue Scale.

**Table 3:** Descriptive statistics and multiple comparisons for the change in variables assessed. Values are expressed as mean ± SD and median (IQR).

Outcome	EQ-5D-5L, Index (MCID = 0.262)		EQ-5D-5L, VAS (MCID = 7.5)		Between-Group Differences *p*-Value; *r* Effect-Size (a) MCID for EQ-5D-5L, Index (b) MCID for EQ-5D-5L, VAS
	Not Exceeded MCID	Exceeded MCID	Not Exceeded MCID	Exceeded MCID	
Health-related quality of life					
EQ-5D-5L (index)	— —	— —	0.121 ± 0.188 0.128 (0.021–0.219)	0.209 ± 0.158 0.218 (0.09–0.302) ^a^	(a)— (b) *p* = 0.200; *r* = 0.20
EQ-5D-5L (VAS)	13.3 ± 8.9 15 (7.5–20) ^a^	26.4 ± 14.9 27.5 (20–35) ^a^	— —	— —	(a) *p* = 0.002; *r* = 0.47 (b)—
Inspiratory Muscle Function					
MIP (cmH_2_O)	26.9 ± 15.5 25 (17–37) ^a^	45.4 ± 19.8 46 (34–51) ^a^	12.5 ± 6.8 12.5 (10–18) ^a^	37.9 ± 17.7 35 (27–50) ^a^	(a) *p* = 0.004; *r* = 0.44 (b) *p* < 0.001; *r* = 0.60
MIP (% pred)	26.3 ± 16.3 23.5 (16.4–35.5) ^a^	42.5 ± 15.2 45.2 (32–52.6) ^a^	11.5 ± 7.15 10.9 (7–17.3) ^a^	36.5 ± 15.8 35.5 (23.8–51.5) ^a^	(a) *p* = 0.004; *r* = 0.44 (b) *p* < 0.001; *r* = 0.60
IME (sec)	240.4 ± 151.4 246.5 (144–354) ^a^	337.1 ± 156.8 361 (162–457) ^a^	125.2 ± 126.6 121 (30–237.5) ^a^	307.3 ± 145.5 306.5 (210–428) ^a^	(a) *p* = 0.083; *r* = 0.27 (b) *p* = 0.003; *r* = 0.45
Exercise tolerance (Ruffier index)	−1 ± 2.3 −1.2 (−2.6–0.1) ^b^	−1.6 ± 3.3 −2 (−2.7–0.7)	0.3 ± 1.9 −0.1 (−0.7–1.7)	−1.6 ± 2.7 −2.1 (−3–0) ^a^	(a) *p* = 0.759; *r* = 0.05 (b) *p* = 0.032; *r* = 0.33
Peripheral muscle strength					
1 min STS (n of squats)	10 ± 9.6 11.5 (4–16.5) ^a^	16.3 ± 10.1 16 (9–22) ^a^	6.4 ± 13.5 5.5 (2–15.5)	13.5 ± 8.8 13 (8–17) ^a^	(a) *p* = 0.092; *r* = 0.26 (b) *p* = 0.023; *r* = 0.35
Handgrip (Kg)	−0.6 ± 4.1 −1 (−3.7–2)	3.5 ± 4.8 2.7 (2–5) ^b^	0 ± 4.2 −0.7 (−2.7–4.2)	0.9 ± 4.8 1.2 (−2.5–3)	(a) *p* = 0.004; *r* = 0.44 (b) *p* = 0.898; *r* = 0.02
Lung function					
FVC (% pred)	2.8 ± 11.1 1 (−2.5–7.5)	4.4 ± 12.5 3.5 (1–9) ^b^	0.7 ± 15.2 −1 (−4.5–6.5)	3.9 ± 10.6 2.5 (0–8) ^b^	(a) *p* = 0.285; *r* = 0.16 (b) *p* = 0.255; *r* = 0.18
FEV_1_ (% pred)	3.4 ± 10.1 2.5 (−1.5–6.5) ^b^	−1.1 ± 10.3 −2 (−5–5)	1.1 ± 14.7 0 (−4–3)	2.1 ± 9.2 2 (−3–6)	(a) *p* = 0.173; *r* = 0.21 (b) *p* = 0.386; *r* = 0.13
FEV_1_/FVC (%)	−0.7 ± 3.2 −1 (−2–1)	−1.6 ± 2.6 −1.5 (−3–0)	0 ± 2.7 0 (0–1.5)	−1.3 ± 3.1 −1 (−2–0) ^a^	(a) *p* = 0.306; *r* = 0.16 (b) *p* = 0.052; *r* = 0.30

Abbreviations: EQ-5D-5L, EuroQol-5D questionnaire; HRQoL. Health-related quality of life; IME, inspiratory muscle endurance; MCID, minimal clinically important difference; MIP, maximal inspiratory pressure; FEV1, force expiratory volume 1st second; FVC, force vital capacity; STS, sit-to-stand; VAS, visual analogue scale; % pred, percentage of predicted value. ^a^ Statistically significant within-group differences from baseline values, *p* < 0.05. ^b^ Statistically significant within-group differences from baseline values, *p* < 0.01.

## Data Availability

Available upon request.
